# Metabolism Pathway Blood Proteomic Differences in Lewy Body Dementia Compared to Alzheimer’s Disease

**DOI:** 10.3390/ijms27156875

**Published:** 2026-07-31

**Authors:** Ariana N. Pritha, Leonidas Chouliaras, Peter Swann, Maria Prats-Sedano, Anna McKeever, Amanda Heslegrave, Nicholas J. Ashton, Henrik Zetterberg, Li Su, Maura Malpetti, James B. Rowe, John T. O’Brien

**Affiliations:** 1 Department of Psychiatry, School of Clinical Medicine, University of Cambridge, Cambridge CB2 0SP, UK; anp47@cam.ac.uk (A.N.P.); lc716@medschl.cam.ac.uk (L.C.); ps689@medschl.cam.ac.uk (P.S.);; 2 Specialist Dementia and Frailty Service, Essex Partnership University NHS Foundation Trust, St Margaret’s Hospital, Epping CM16 6TN, UK; 3 Dementia Research Institute, University College London, London NW1 3BT, UK; a.heslegrave@ucl.ac.uk (A.H.);; 4 Department of Neurodegenerative Disease, UCL Queen Square Institute of Neurology, London WC1N 3BG, UK; 5 Department of Psychiatry and Neurochemistry, University of Gothenburg, 405 30 Gothenburg, Sweden; 6 Banner Alzheimer’s Institute, University of Arizona, Phoenix, AZ 85004, USA; 7 Banner Sun Health Research Institute, Sun City, AZ 85351, USA; 8 Clinical Neurochemistry Laboratory, Sahlgrenska University Hospital, 413 45 Mölndal, Sweden; 9 Hong Kong Center for Neurodegenerative Diseases, Clear Water Bay, Hong Kong, China; 10 Wisconsin Alzheimer’s Disease Research Center, School of Medicine and Public Health, University of Wisconsin-Madison, Madison, WI 53706, USA; 11 Department of Clinical Neurosciences, School of Clinical Medicine, University of Cambridge, Cambridge CB2 0SP, UK; 12 Dementia Research Institute, Cambridge CB2 0AH, UK; 13 UK Dementia Research Institute at Cambridge, University of Cambridge, Cambridge CB2 0SP, UK; 14 Medical Research Council Cognition and Brain Sciences Unit, Cambridge CB2 7EF, UK

**Keywords:** Dementia with Lewy Bodies (DLB), protein blood biomarkers, proteomics, Alzheimer’s disease

## Abstract

Dementia with Lewy Bodies (DLB) is the most common neurodegenerative dementia after Alzheimer’s disease (AD), but it remains challenging to diagnose due to overlapping symptoms and mixed pathologies. This pilot study tested whether DLB has different metabolic blood proteomic profiles compared to AD and controls. Serum was analysed from people with DLB (*n* = 20), a group with AD (either with Alzheimer’s disease dementia or Mild Cognitive Impairment with a positive amyloid positron emission tomography scan (MCI+/AD) (*n* = 15), and similarly aged controls (*n* = 15) using the Olink Metabolism panel encompassing 92 proteins. Six proteins (PILRB, LRIG1, NECTIN2, TINAGL1, SSC4D, and FKBP4) were significantly different in DLB compared with the controls, and one protein (SERPINB8) was differentially expressed when compared with MCI+/AD. Receiver operating characteristic curves for biologically relevant proteins that have previously established roles in neurodegenerative and cognitive properties showed that RNASE3 levels could differentiate DLB from MCI+/AD (area under the curve (AUC) = 0.717, sensitivity = 0.850, and specificity = 0.600). A multimarker model incorporating RNASE3 with phosphorylated tau 217 (pTau217) and polygenic risk scores for LBD achieved improved accuracy in discriminating DLB from MCI+/AD (AUC = 0.963, sensitivity = 1.000, and specificity = 0.900). Pathway analyses revealed dysregulation in cortisol signalling, inflammation resolution, and ErbB4-mediated neuroplasticity, which point towards peripheral protein alterations related to the adaptation to stress, immune regulation, and synaptic integrity in DLB. The present exploratory study highlights potential pathophysiological mechanisms implicated in DLB, suggesting that a multimodal biomarker panel may perform better compared to single proteins. Considering the small sample sizes, the findings will need to be replicated in larger cohorts.

## 1. Introduction

Dementia with Lewy bodies (DLB) is a common cause of dementia in older people, accounting for up to 15–20% of all dementia cases [1]. DLB is underdiagnosed and has higher care costs than Alzheimer’s disease (AD) [2]. It is associated with a poorer outcome than AD, with both more rapid progression and higher mortality [3,4]. In line with progress in AD fluid biomarkers that help improve diagnosis, there is an urgent need for clinically accessible, accurate blood biomarkers that can support DLB diagnosis and monitoring of disease progression, while also shedding light on the underlying pathophysiology.

In recent years, the measurement of AD-related neuropathology markers, such as amyloid beta and phosphorylated tau in cerebrospinal fluid and blood, has reached clinical practice for diagnostic purposes [5]. Nevertheless, findings on blood biomarkers for synucleopathies have not yet reached the standards required in order to be implemented in clinical practice [6,7,8,9]. Seed amplification assays (SAA) for α-synuclein in cerebrospinal fluid (CSF) have been developed as a biomarker to help establish a diagnosis and are on the path for implementation in routine clinical care [10]. Attempting to translate this for blood has been more difficult as there is, overall, less protein in blood, especially in plasma [7]. Dopa decarboxylase (DDC) has been demonstrated to potentially be a biomarker for DLB in CSF [11]. DDC levels in blood, however, are strongly influenced by dopaminergic medications that many patients with Parkinsonism are prescribed [12]. Currently, there is not a single widely adopted blood biomarker that directly and robustly captures α-synuclein pathology with the same maturity as CSF SAA, and blood-based approaches face matrix effects (e.g., inhibitory plasma proteins) and remain an active area of optimization/validation rather than the standard care [13].

Due to individuals varying substantially in presentation, co-pathology, and rates of progression, high-throughput proteomics has become a practical way to move beyond single-analyte biomarkers and instead capture pathway-level dysregulation (e.g., synaptic biology, myelination, complement/innate immunity, and vascular signalling) that cuts across clinical neurodegenerative syndromes. Across the broader literature, proteomics approaches in AD and DLB generally fall into two complementary categories: (1) antibody/affinity-based multiplex platforms (e.g., proximity extension assays) that offer scalable, sensitive profiling across hundreds to thousands of analytes, and (2) mass spectrometry (LC–MS/MS) workflows that are less constrained by predefined targets and can provide orthogonal validation/discovery [14,15,16].

A major driver of this shift has been the Olink’s Proximity Extension Assay (PEA) platform, which uses paired antibody recognition coupled to DNA tags to generate a quantitative sequencing/qPCR readout with high multiplexing at a low sample volume [15,17]. Given that DLB is linked to systemic disturbances in energy, lipid, and glucose metabolism that can be detected in peripheral blood [18,19], a targeted Olink Metabolism panel provides a sensitive way to screen a broad range of metabolism-related proteins. Previous studies on CSF using the Olink Metabolism Panel have identified Dopa Decarboxylase (DDC) as a potential fluid marker of synucleopathies like DLB [20,21]. Olink assays are commonly deployed either as targeted panels (e.g., Target 96 focused panels), and they will be the focus of this study, aiming to expand on previous studies using this panel or discovery-scale platforms (e.g., Explore), both of which provide outputs as NPX (Normalised Protein Expression)—relative, log2-scaled values designed for cross-sample comparability after normalisation and QC [17]. The UK Biobank Pharma Proteomics Project (UKB-PPP) illustrates the population-scale value of these panels for discovery and translation. In the pilot release, plasma proteomics were generated in ~54,000 UK Biobank participants using Olink Explore 3072, quantifying ~2941 analytes (~2923 unique proteins) and enabling large-scale analyses connecting protein levels to genotypes, disease phenotypes, and drug-target biology [5,22]. Studies using the UK Biobank data highlighted a key point that brain-derived plasma proteins not only were indicative of brain age but also driven by proteins such as NEFL, MOG, GFAP, BCAN, and PTPRR, mapping mainly to oligodendrocyte-lineage, neuronal, and astrocytic genes and enriched for perineuronal net and white-matter–related pathways [23,24].

A key gap in the field is how to integrate biomarker approaches so that panels can be used not only to distinguish dementia subtypes but also to classify the stage of disease in individual patients. Building on previous data in the field, in this pilot study, the primary aim was to identify potential blood serum biomarkers for DLB compared to controls and AD using the Olink Target Metabolism panel. The secondary aim was to test whether a combination of markers including proteomic markers along with more established markers, such as phosphorylated tau in plasma and polygenic risk scores, can improve outcomes compared to single markers alone.

## 2. Results

### 2.1. Differential Protein Expression in DLB vs. Control and DLB vs. MCI+/AD

**Figure 1:**
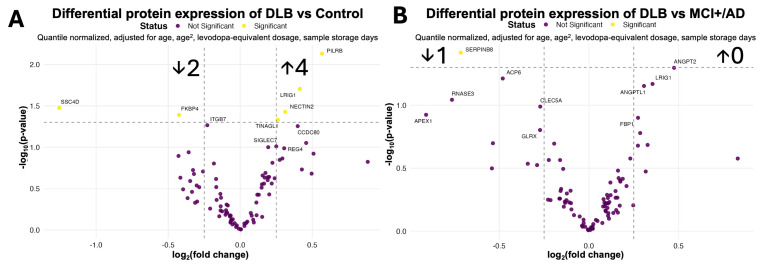
The *Y*-axis is –log_10_(*p*-value) and the *x*-axis demonstrates the log_2_(fold-change). The yellow markers are p-adjusted significant values, and the purple circles are not significant based on the set thresholds of *p* < 0.05 and |log_2_(fold-change)| > 1.25. (**A**) Volcano plot of Lewy Body Dementia vs. control contrasted. (**B**) Volcano plot of Lewy Body Dementia vs. MCI+/AD.

The volcano plot was processed using quantile normalization across samples from the Olink data. There was a covariate adjustment for age, sex, levodopa-equivalent dose, sample storage days, nonlinear (Age^2^) term, and low-variance filtering, and an empirical Bayes shrinkage was applied. We reported the *p*-values from the Limma pipeline to encapsulate the biologically small differences. From the thresholds of log2FC > |0.25| and *p* < 0.05, there were two downregulated proteins and four upregulated proteins in the DLB group when compared to the control group. The two downregulated proteins were SSC4D (log2FC = −1.25, *p* = 0.034) and FKBP4 (log2FC = −0.42, *p* = 0.041) while the four upregulated proteins were PILRB (log2FC = 0.56, *p* = 0.0074), LRIG1 (log2FC = 0.41, *p* = 0.020), NECTIN2 (log2FC = 0.31, *p* = 0.038), and TINAGL1 (log2FC = 0.26, *p* = 0.047) (Figure 1A). When we compared DLB to AD, based on the thresholds of log2FC > |0.25| and *p* < 0.05, SERPINB8 (log_2_FC = −0.71, *p* = 0.039) was the only protein significantly dysregulated, and interestingly, DDC did not pass the DE-protein threshold (Figure 1B).

### 2.2. Classification Analysis Using Receiver Operating Characteristic Curves for Differentiating DLB from MCI+/AD

**Figure 2:**
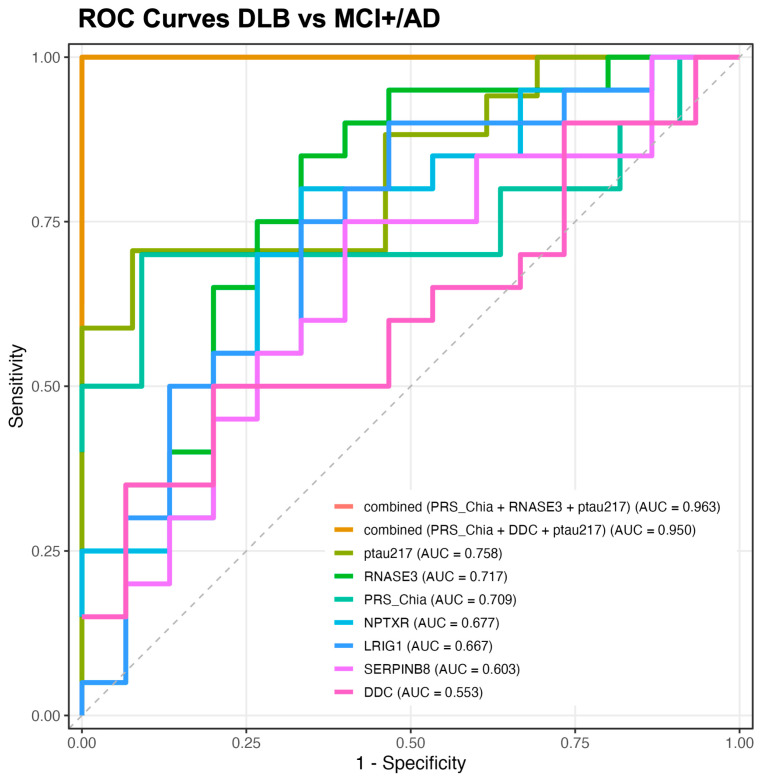
The ROC plot shows the top protein markers with the highest/of interest AUCs, polygenic risk score (PRS), and combinations of markers. The plot demonstrates the classification performance of individual protein biomarkers and polygenic risk scores (PRS) in differentiating Lewy Body Dementia (DLB) from MCI+/AD with and without APOE. Individual markers including pTau217 (AUC = 0.76), RNASE3 (AUC = 0.72), NPTXR (AUC = 0.68), and LRIG1 (AUC = 0.67) showed moderate diagnostic power.

As reported previously [25], p-tau217 had the strongest performance for differentiating DLB from MCI+/AD [AUC = 0.758 (95% CI 0.569–0.947), threshold = 0.695, sensitivity = 0.706, and specificity = 0.846], while PRS_LBD no *APOE*_, alone demonstrated different results [AUC = 0.709 (95% CI 0.449–0.969), threshold = 0.32, sensitivity = 0.700, and specificity = 0.818]. To expand beyond proteins identified through differential expression alone, we selected a small set of biologically informed candidate markers based on predefined criteria. Proteins were prioritised if they met at least one of the following conditions: (i) previous association with cognitive decline or neurodegeneration in human proteomic studies, (ii) evidence of involvement in synaptic function, neuroinflammation, or Lewy body disease biology, or (iii) prior reporting as a biomarker candidate in DLB, Parkinson’s disease, or Alzheimer’s disease. Using these criteria, RNASE3, NPTXR, LRIG1, SERPINB8, and DDC were selected for an exploratory ROC analysis. RNASE3 and NPTXR have been linked to cognitive performance and neurodegenerative processes, and LRIG1 emerged as one of the strongest differentially expressed proteins in the present dataset. SERPINB8 was the only protein significantly different between DLB and MCI+/AD, and DDC was included because previous CSF proteomic studies have identified it as a promising marker of Lewy body disorders, despite not reaching significance in the present serum cohort. Looking at the single protein markers, RNASE3 showed an AUC = 0.717 (95% CI 0.526–0.908, threshold = 3.222, sensitivity = 0.850, and specificity = 0.600), NPTXR an AUC = 0.677 (95% CI 0.488–0.865, threshold = 3.349, sensitivity = 0.800, and specificity = 0.600), and LRIG1 an AUC = 0.667 (95% CI 0.471–0.863, threshold = 3.644, sensitivity = 0.900, and specificity = 0.467) (Figure 2).

Additional markers performed less optimally, including SERPINB8 (AUC = 0.603, 95% CI 0.407–0.799, threshold = 2.316, sensitivity = 0.750, and specificity = 0.533), which was demonstrated to be significantly dysregulated in a previous analysis (Figure 2) and DDC (AUC = 0.553, 95% CI 0.357–0.750, threshold = 5.297, sensitivity = 0.500, and specificity = 0.733) was reported to be a differentiator of DLB in cerebrospinal fluid when upregulated [12].

Multimarker models markedly improved discrimination, with PRS_LBD no *APOE*_ + RNASE3 + p-tau217 achieving the highest accuracy (AUC = 0.963, 95% CI 0.881–1.000, threshold = 0.338, sensitivity = 1.000, and specificity = 0.900) (Figure 2).

### 2.3. Serum Proteins Associated with Survival in the DLB and MCI+/AD Group

**Figure 3:**
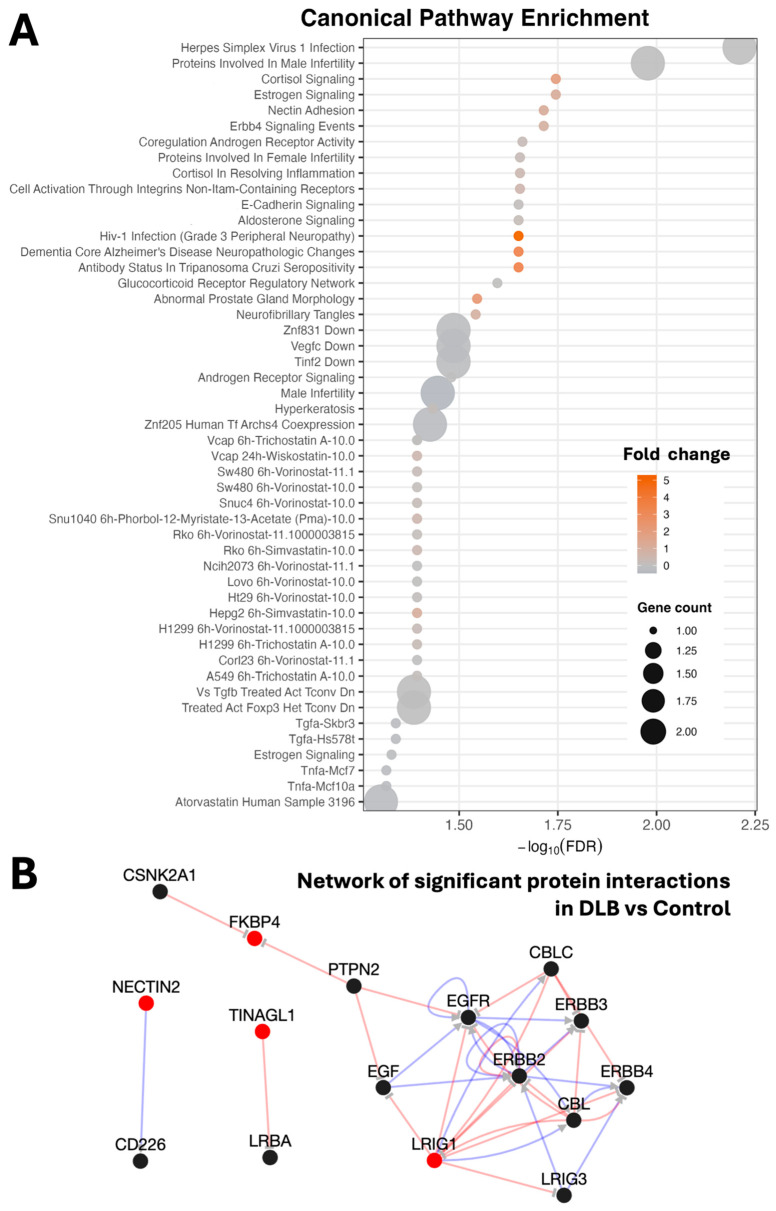
(**A**) Network of common proteins based on three Limma pipeline differential expression analyses—comparisons of DLB vs. MCI+/AD, DLB vs. control, and MCI+/AD vs. control. The blue lines are indicative of upregulation and the red lines are downregulated. The thresholds of the molecules—all *p*-values of <0.05—were used to identify common/unique markers between the different groups. The absolute log2FC can be determined by the increasing sizes of circles. Using all available human gene datasets, the canonical pathways are represented for the six dysregulated proteins, with *p* < 0.05 and log2FC > abs.0.25. There are pathways of dementia core, cortisol signalling, neurofibrillary tangles, and more listed through the graph. (**B**) A network using Konnect2prot 2.0, where the blue lines indicate downregulation and the red lines indicate upregulation. The arrows demonstrate the direct effect relationship and the perpendicular lines indicate inhibition. The red circles are proteins input into the online software and the black circles are established interacting proteins between the proteins of interest.

Protein expression data in combination with survival data, with the greatest difference between the DLB and MCI+/AD groups and therefore the strongest association with survival, include METRNL1 (log(hazard ratio) difference = 2.21), ROR1 (log(hazard ratio) difference = 2.07), and GFAP (log(hazard ratio) difference = 1.97), and these are more elucidated in Figure 3.

### 2.4. Network and Pathway Analysis of Differentially Expressed Proteins in Controls, MCI+/AD, and DLB

Identifying which pathways are most enriched using the differentially expressed proteins and retrieving significant pathways from all gene and protein databases through ShinyGo 0.85.1 provides insight into the coordinated biological processes that may be disrupted due to DLB (Figure 3A). For example, seeing enrichment in immune, metabolic, and neuronal signalling pathways suggests that the observed protein changes are part of a larger, regulated system responding to disease pathology mechanistic functions. The protein network comparison generated using the Konnect2Prot 2.0 platform demonstrates how there are 12 interacting proteins, with 4 of the 6 DE proteins of interest, with an exploratory possibility of being related to proteins that may not have been assessed through the panel (Figure 3B). Mechanistic explanations for protein dysregulation can elucidate how protein expression levels are associated andprovide an avenue for exploring how a panel of proteins may profile DLB.

## 3. Discussion

This study has shown that, using a panel of blood biomarkers, DLB has a different peripheral blood proteomic signature compared to both healthy controls and people with MCI+/AD. It also showed specific proteins associated with survival rates in DLB that could serve as prognostic markers and could be used to identify protein pathways that are dysregulated in DLB.

LRIG1 appeared among the top upregulated signals, and additional immune/glial-associated proteins (e.g., PILRB and TINAGL1) were elevated when comparing DLB to controls (Figure 1A). The LRIG1 gene has literature connecting it to dementias [10,26,27,28], immune mechanisms [29], and neurogenesis [30,31,32]. LRIG1 appears to be a multifunctional player in neural structure and immune regulation within the context of neurodegenerative disorders, especially as it may mediate synaptic reorganisation with neuroinflammatory responses pertaining to DLB and AD. Recent evidence suggests that LRIG1 can be functionally modulated peripherally (e.g., anti-LRIG1, aka GTC 310-01 monoclonal antibody that targets LRIG1 without crossing the blood-brain barrier), functionally regulates T regulatory (Treg) cells, and provides a novel immunotherapeutic target in addressing AD-like pathology [27]. In the central nervous system, LRIG1 is an intrinsic regulator of the dendritic structure of hippocampal neurons, where LRIG1 loss increases dendritic branching in a BDNF-TrkB hyperactivation process [26], while LRIG1 overexpression decreases dendritic complexity at synapses. In addition, LRIG1 loss supports the proliferation of GABAergic for glutamatergic neural precursor cells through FGF2/IL6/JAK2/STAT3 signalling [33], thus impacting cortical cell infrastructure and neuronal subtype. Taken together, this work indicates that LRIG1 loss or dysregulation may dysregulate neuronal connectivity and the neuroimmune response, which may underlie cognitive decline and neural vulnerability in DLB. Both PILRB and TINAGL1 were elevated in DLB in comparison to the control groups (Figure 1A), suggesting that neuroinflammation and neurodegenerative pathways are implicated in DLB pathophysiology. PILRB is an immunoregulatory receptor that has been previously genetically associated with Alzheimer’s disease (AD) risk loci (ZCWPW1-PILRB) and was found to be elevated in cerebrospinal fluid in patients with AD and dementia, linking it to microglia regulation and immune activation [34]. The upregulation of PILRB seen in DLB may also reflect neuroinflammation and increases in immune signalling and/or a decrease in the clearance of pathologic aggregates. Additionally, TINAGL1 is a secreted matricellular protein that is predominantly expressed by reactive astrocytes and has been shown to mediate the degeneration of dopaminergic neurons via mitochondrial ROS signalling in biological systems involving mice [35]. TINAGL1 levels are also increased in the cerebrospinal fluid and tissues of individuals with AD and inflammatory conditions, supporting its link to astrocyte-mediated neurotoxicity and chronic activation of the ERK pathway [36]. Together, the increase in both PILRB and TINAGL1 implicates both immune responses and glial stress, which represents a pathological signature in DLB and may reflect the role of both PILRB and TINAGL1 as potential markers of neuroimmune dysregulation and neuronal vulnerability across a spectrum of neurodegenerative diseases.

The proteins FKBP4, SSC4D, and NECTIN2 also resulted from the DLB vs. control comparison, and these proteins are suggestive of stress adaptation, immune modulation, and synapse integrity. FKBP4 is a chaperone involved with the regulation of glucocorticoid and oestrogen receptors that likely play a role in neuroendocrine stress response and protein quality control processes related to Lewy body pathology [37]. SSC4D, a scavenger receptor family member, aligns well with evolving evidence of immune dysregulation and activation of microglia within DLB and has been reported to be associated with all-cause dementia [38]. NECTIN2 is an adhesion molecule that is linked to synaptic connectivity as well as dementia [39], and it indicates possible changes to cell–cell signalling.

DLB compared to AD produced modest differences, suggesting some proteins (e.g., SERPINB8) may reflect shared neurodegenerative stress/proteostasis rather than DLB-specific biology (Figure 1B). Pathway/network analyses converged on stress-hormone/immune signalling and receptor-tyrosine-kinase axes (including EGFR/ERBB-related regulation) that were consistent with a model where systemic immune regulation and synaptic maintenance programs diverge across DLB and AD.

The only protein that was altered in both DLB and MCI+/AD (compared to the controls) was SERPINB8 (Figure 1B), indicating that it may not be a disorder-specific protein but rather a common protein across neurodegenerative processes [40] and possibly as a more generalised neurodegenerative process compared to others [41]. SERPINB8 is part of the serine protease inhibitor family and is involved in the temporal regulation of proteolytic activity in the event of neuroinflammation and cellular stress, which is necessary for the maintenance of proteostasis; however, the consistency in expression between DLB and AD suggests that serpins may reflect a common burden of neuroinflammation and not unique molecular signatures that indicate novel diseases.

Taken together, these profiles reveal that while DLB and AD share core pathways of inflammation, oxidative stress, and proteostasis, they likely diverge in how these processes interact with synaptic and receptor-mediated signalling networks.

In terms of available literature in the field looking at other available proteomic panels, Durcan et al. implemented Alamar’s ultra-sensitive NULISA CNS (~120) + inflammation (250) panels across AD, DLB, frontotemporal dementia, and progressive supranuclear palsy, and they found both disease-specific signals and transdiagnostic prognostic markers (e.g., NfL) [42]. Many of the differentially expressed proteins in our study, like LRIG1, PILRB, TINAGL1, SERPINB8, FKBP4, SSC4D, and NECTIN2, are not included on the Alamar panel and therefore direct comparisons cannot be implemented (Supplementary File S1, NULISA Target Panels). In a recent CSF proteomics study, Del Campo et al. measured ~665 proteins, identified DLB-associated alterations enriched in myelination-related pathways, and derived protein panels that distinguish DLB from AD with high accuracy [20]. Of the chosen profiled proteins in the Olink metabolic panel, only DDC, ENTPD5, FAM3C, KLK10, NPDC1, RTN4R, and SDC4 were the same as the DE-proteins in the Del Campo study. DDC was the only common one between the datasets, but it suggests a future direction of identifying neuroinflammatory metabolic proteins for further assay studies on similar cohorts.

Regarding the multimarker combination models incorporating established PRS and ptau biomarkers, McKeever et al. showed that combining PRS for AD (including *APOE* risk score and AD PRS excluding *APOE*) with plasma p-tau181 modestly improves diagnostic classification across controls, DLB, and MCI+/AD (with AUCs in the ~0.71–0.82 range depending on contrast/model). We extended these findings by examining the combination of p-tau217 with the LBD PRS, both with and without *APOE* for DLB-relevant proteomic features, to test whether multi-marker models better separate DLB from AD and stratify co-pathology [43]. Interestingly, the LBD PRS without *APOE* demonstrated the strongest AUC, but both combined models fared well through this analysis.

**Figure 4:**
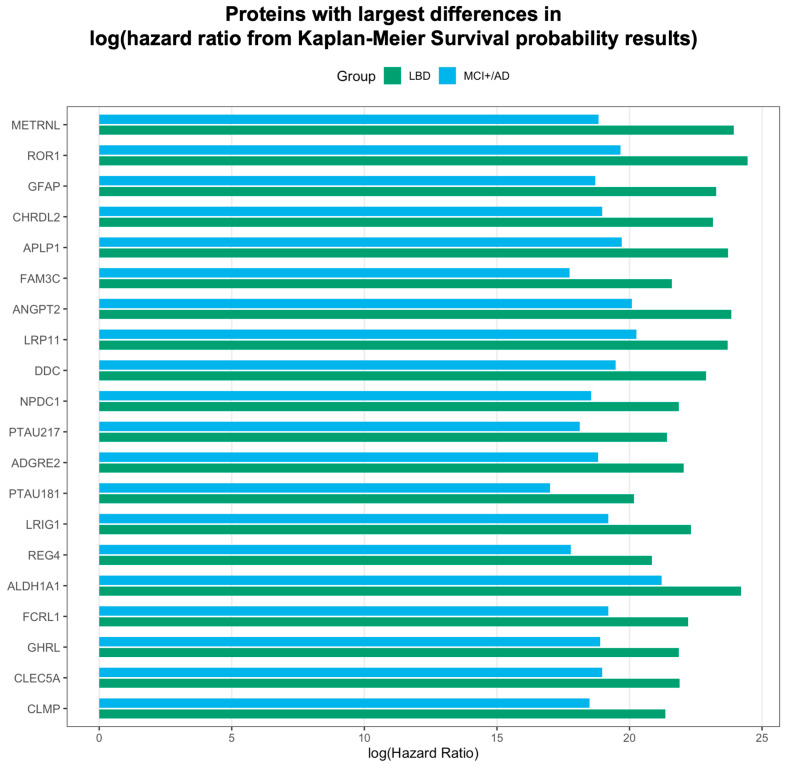
Hazard ratios were implemented from the Kaplan–Meier Survival probability results and analysed in relation to each protein for the disease conditions for the DLB and MCI+/AD groups, and those with the highest log_10_(Hazard Ratio) were plotted. Protein markers with the greatest difference in hazard ratio between DLB and MCI+/AD are first in descending order. Proteins associated with a higher risk of DLB compared to MCI+/AD include DDC, LRIG1, GFAP, and others.

Using age as the underlying time scale, Kaplan–Meier curves showed broadly overlapping survival in the LBD and MCI+/AD groups over the available follow-up. Consistent with this, Cox models adjusting for sex did not identify a significant overall effect of diagnostic group on mortality risk (Figure 1). Exploratory protein-wise Cox models revealed a small subset of analytes (e.g., METRNL, ROR1, GFAP, APLP1, ANGPT2, and LRP11) with larger positive log hazard ratios in the LBD group than in MCI+/AD (Figure 4), suggesting potential group-specific mortality signatures.

In terms of pathway analyses, the enrichment of canonical pathways related to cortisol signalling, glucocorticoid receptor regulation, and ErbB4 signalling underscores the complex interaction between stress response, neuroinflammation, and synaptic maintenance in DLB (Figure 3A). Cortisol and its downstream glucocorticoid receptor (GR) network normally act to resolve inflammation; however, chronic activation of the hypothalamic–pituitary–adrenal (HPA) axis can lead to GR resistance, sustained inflammatory signalling, and neuronal vulnerability. The detection of Dementia Core Alzheimer’s Disease Neuropathologic Changes and Neurofibrillary Tangles pathways further supports overlap between DLB and AD pathology, where stress-mediated dysregulation contributes to amyloid processing, tau phosphorylation, and impaired clearance mechanisms. ErbB4 signalling, critical for dendritic maintenance and cortical connectivity, may act in parallel with glucocorticoid pathways to influence neuronal plasticity and degeneration. Peripheral and systemic processes, such as infertility-related signalling and grade 3 peripheral neuropathy, point to broader neuroendocrine and inflammatory disturbances that extend beyond the central nervous system. Collectively, these findings suggest that chronic stress and receptor signalling dysregulation converge on pathways that bridge neuroimmune dysfunction, synaptic loss, and systemic inflammation, offering insight into shared mechanisms of neurodegeneration across dementia subtypes.

The interaction network in Figure 3B shows that multiple identified proteins converge on the EGFR/ERBB signalling axis, a pathway that has previously only been examined in the context of oncogenesis but is beginning to be touched on regarding neurodegenerative processes. EGFR and its dimerisation partners, ERBB2–4, regulate cellular stress response, survival, and maintenance of synapses, but when dysregulated, they may contribute to the loss of autophagic clearance and accelerate the aggregation of α-synuclein, which is a hallmark of DLB [44]. Frost regulatory proteins such as CBL/CBLC and LRIG3, which modify the turnover of EGFR/ERBB receptors, provide another of many examples of how feedback control is disrupted to sustain aberrant signalling and widespread chronic neuroinflammation [45,46,47]. In addition to these proteins, peripheral modulators (e.g., EGF and immune-related proteins (e.g., CD226 and LRBA)) support the notion of crosstalk and the support of ongoing neuroinflammatory processes. Direct experimental testing to corroborate this in DLB remains limited; however, protein–protein interactions in the network provide a key resource to support cellular vulnerability commonalities between disrupted receptor signalling, proteostasis support, and cell repair mechanisms [48]. Collectively, these factors lend support for EGFR–ERBB pathway components to be included in future multiplex protein panels, specifically as these factors may improve biomarker sensitivity due to their involvement in measuring coordinated dysregulation between growth factor and immune signalling in DLB. These findings may support translational approaches by identifying protein signatures that could improve DLB diagnosis, subtype classification, and patient stratification for clinical trials. The Olink Metabolism panel can further be developed for clinical testing, as any candidate biomarker signature would still require validation in larger independent cohorts. In the future, combining Olink proteomic data with genetic and DNA methylation profiles may provide a more accurate multi-omic approach to distinguish DLB from related neurodegenerative disorders.

### Limitations

This pilot study had a limited cohort size, and group imbalances in sex distribution may have diminished the possibility of determining sex differences, but it may also be an intrinsic signature in DLB cohorts. Levodopa-equivalent dosage was controlled in the analyses, but due to the small sample size and uneven numbers between sexes, the skew of diseased populations, which coincide with the drug-dosed populations, could confound protein expression profiles in the DLB group. Additionally, the available dataset did not permit stratification of DLB cases by amyloid status (amyloid-positive versus amyloid-negative), precluding the assessment of intra-diagnostic heterogeneity. Currently, there are many databases on AD-associated pathways, though DLB has been under-investigated. Future studies with larger, demographically balanced cohorts and standardised medication data will be essential to validate and extend these preliminary observations.

## 4. Materials and Methods

### 4.1. Participants and Clinical Data

All participant-collected data were derived from the NIMROD study cohort, whose full inclusion and exclusion criteria are outlined in the published study protocol [49]. Healthy controls and participants with MCI, AD, or DLB were included based on the relevant clinical diagnostic criteria (National Institute on Aging-Alzheimer’s Association Criteria, 2005/2017 McKeith consensus criteria for DLB) as previously described [50]. MCI PET-Aβ-positive participants were defined by the presence of a clinical diagnosis of MCI (subjective memory reports supported by objective evidence of impairments, but no impairment in activities of daily living) and amyloid positivity using Pittsburgh Compound B (PIB) PET using a cut-off point of 19 in the centiloid scale.

### 4.2. Clinical Assessments

As reported in our group’s prior studies, at enrolment, all participants received a detailed clinical evaluation that included cognitive and neuropsychiatric testing. Follow-up assessments were then carried out annually for up to 3 years as part of the NIMROD study [49]. Cognitive performance was quantified using the Addenbrooke’s Cognitive Examination–Revised (ACE-R) [50]. Survival data were obtained from linked electronic patient care records that were available for the MCI+/AD and the DLB groups. There was a total of 22 deceased patients based on records across groups. Details of the demographics, cognitive scores, and survival rates of participants are presented in Table 1.

### 4.3. Sample Preparation & Molecular Analyses

Venous blood (≈80 mL) was collected via venipuncture. The samples were centrifuged to generate serum and plasma, aliquoted, and stored at −70 °C for later batch testing.

### 4.4. Assays

Blood serum samples from people with DLB (*n* = 20), people with MCI+/AD (*n* = 15), and controls (*n* = 15) were analysed with the Olink Target 96 Biological Process—Metabolism panel (Olink Proteomics AB, Uppsala, Sweden) containing 92 different target proteins (the list and details of the proteins can be found in Supplementary File S1, Olink Metabolism Panel). The proteomic analysis was carried out at the UK Dementia Research Institute (DRI) Biomarker Factory at University College London (UCL). Plasma pTau217 was quantified at the Clinical Neurochemistry Laboratory in Mölndal (Sweden) with the ALZpath ptau217 assay [51]. Biomarker values were log-transformed to improve normality and standardised (using the whole-sample mean and SD) for modelling [43]. Genetic data were generated using the Illumina OmniExpress-24 v1.3 microarray and quality controlled as previously described [43]. Summary statistics from a published LBD genome-wide association study [52] were used as the discovery dataset, and genetic risk scores (PRS) were computed in our independent cohort as a weighted sum of risk alleles, with SNP weights given by the GWAS effect sizes. Five SNPs (in *APOE*, *GBA*, *SNCA*, *BIN1*, and *TMEM175*) meeting genome-wide significance in the discovery GWAS were included in PRS_LBD_ as reported in Table 1 by Chia et al. [52]. Additional scores excluding *APOE* variants were calculated to evaluate non-*APOE* genetic risk (PRS_LBD no *APOE*_) (Supplementary File S1). Genetic risk scores were standardized within-sample.

### 4.5. Statistical & Bioinformatics Analyses

All analyses were conducted in R (v4.3). Protein NPX values were first quantile-normalised and residualised to account for relevant covariates, including age [53], sex [54], sample storage days [55], and Levodopa-equivalent dose [56], using linear modelling because of their possible effect on protein levels [57]. These residualised NPX values were used for downstream group comparisons and visualisation.

The Limma package was applied to identify significantly dysregulated proteins between diagnostic contrasts (DLB vs. control, MCI+/AD vs. control, DLB vs. MCI+/AD). Models were adjusted for age, age^2^ [58], sex [54], Levodopa-dose [59], and storage days [55], and empirical a Bayes moderation was used to stabilize the variance estimates. Volcano plots were generated to visualise the fold changes and significance thresholds (|log_2_FC| ≥ 0.25, FDR < 0.05). We evaluated whether a nonlinear age term was warranted by comparing a linear-age model to a quadratic-age model. The age^2^ term showed evidence of association across proteins (n_FDR < 0.05 = 1, minimum FDR = 0.0498, minimum *p* = 5.41 × 10^−4^), and its inclusion modestly altered diagnosis-associated coefficients (absolute ΔlogFC: median 0.0224, mean 0.0259, IQR 0.0125–0.0349, max 0.1417; correlation of Diagnosis logFC estimates between models r = 0.9937). Accordingly, age^2^ was retained in the final adjustment model.

For the ROC analyses, NPX values were first residualised for age, sex, storage days, and Levodopa-equivalent dose using linear regression, and these covariate-adjusted residuals were used as predictors of diagnostic group. Receiver operating characteristic (ROC) curves were computed using the pROC package to evaluate the discriminative performance of the greatest AUC value biomarkers between DLB and MCI+/AD. Individual and combined models (including pTau217 [49,51], biologically relevant protein markers, and PRS) were tested using logistic regression through a multi-parametric model. Candidate proteins for exploratory ROC analyses were selected a priori based on differential-expression results and evidence from the literature supporting associations with cognitive decline, neurodegeneration, synaptic biology, or Lewy Body Disease pathology. Differences between ROC curves were assessed using DeLong’s test for correlated ROC curves implemented in the pROC package. Comparisons were performed between the best-performing single-marker model and the corresponding multimarker models to determine whether the addition of biomarkers significantly improved classification performance.

ShinyGO was used for pathway enrichment across human gene/protein databases, revealing canonical pathways relevant to neurodegeneration, inflammation, and synaptic signalling [60]. Konnect2Prot 2.0 was employed to generate exploratory protein–interaction networks, highlighting direct and indirect molecular relationships among significant proteins [61].

Survival analysis using the Kaplan–Meier model was generated and combined with protein expression levels between the MCI+/AD and DLB groups for comparison (Figure 4).

## 5. Conclusions

From employing exploratory blood proteomics methods, we found that individuals having DLB displayed a distinct peripheral protein pattern that differentiated them from the controls and the MCI+/AD group, where LRIG1 turned out to be the most consistent upregulated signal. The pathway and network patterns indicated a combined disturbance in neuroendocrine stress signalling and immune regulation, which might be indicative of disease-specific inflammatory and synaptic-sustaining programs. However, these initial findings need to be confirmed in larger, well-paired cohorts with uniform biomarker and drug annotation before any conclusion regarding their clinical usefulness can be drawn.

## Figures and Tables

**Table 1:** Subject demographics and summary of ANCOVA stats for DLB vs. MCI+/AD vs. control. Baseline characteristics and survival outcomes. Control (*n* = 15), MCI+/AD (*n* = 15), and DLB (*n* = 20) groups were comparable in age (*p* = 0.205) and sex distribution (*p* = 0.0896). Cognitive performance differed significantly across the groups, with progressively lower MMSE (*p* = 2.89 × 10^−6^) and ACE-R scores (*p* = 3.67 × 10^−8^) observed in MCI+/AD and DLB compared to the controls. Mean survival time was 6.42 years for MCI+/AD and 3.89 years for DLB, with a significant log *p*-rank of <0.0001.

Variable	Control (*n* = 15)	MCI+/AD (*n* = 15)	DLB (*n* = 20)	*p*-Value
Age	68.8 ± 5.5	72.5 ± 9.2	72.8 ± 6.0	0.20423887
Sex	M7/F8	M11/F4	M17/F3	0.089576085
MMSE	28.9 ± 1.1	25.7 ± 2.4	22.4 ± 4.5	2.8803 × 10^−6^
ACE-R	92.1 ± 5.6	79.8 ± 8.1	67.2 ± 13.4	3.6652× 10^−8^
Survival in years (mean)	-	6.42 (*n* = 15, 5 deaths)	3.89 (*n* = 20, 17 deaths)	<0.0001

## Data Availability

The scripts utilised for analysis and presented in this study are available on request from the corresponding author and data are available in the Supplementary File S1 provided.

## Supplementary Materials

The following supporting information can be downloaded at: https://www.mdpi.com/article/10.3390/ijms27156875/s1.
